# Detrimental Effects of Chlorhexidine on Articular Cartilage Viability, Matrix, and Mechanics

**DOI:** 10.1177/03635465241226952

**Published:** 2024-02-14

**Authors:** Maziar Moslehyazdi, Benjamin Bielajew, John A. Schlechter, Jerry C. Hu, Kyriacos A. Athanasiou, Dean Wang

**Affiliations:** * Department of Orthopaedic Surgery, University of California, Irvine, Orange, California, USA; † Department of Biomedical Engineering, University of California, Irvine, Irvine, California, USA; ‡ Pediatric Orthopedic Specialists Orange County, Children’s Hospital of Orange County, Orange, California, USA; Investigation performed at the University of California, Irvine, Irvine, California, USA

**Keywords:** chlorhexidine, cartilage, viability, mechanics

## Abstract

**Background:**

Chlorhexidine gluconate (CHG) solution is commonly used as an antiseptic irrigation for bacterial decontamination during orthopaedic surgery. Although the chondrotoxicity of CHG on articular cartilage has been reported, the full extent of CHG-related chondrotoxicity and its effects on the extracellular matrix and mechanical properties are unknown.

**Purpose:**

To investigate the in vitro effects of a single 1-minute CHG exposure on the viability, biochemical content, and mechanics of native articular cartilage explants.

**Study Design:**

Controlled laboratory study.

**Methods:**

Articular cartilage explants (6 per group) were harvested from femoral condyles of the porcine stifle and sectioned at tidemark. Explants were bathed in CHG solution (0.05% CHG in sterile water) at varying concentrations (0% control, 0.01% CHG, and 0.05% CHG) for 1 minute, followed by complete phosphate-buffered saline wash and culture in chondrogenic medium. At 7 days after CHG exposure, cell viability, matrix content (collagen and glycosaminoglycan [GAG]), and compressive mechanical properties (creep indentation testing) were assessed.

**Results:**

One-minute CHG exposure was chondrotoxic to explants, with both 0.05% CHG (2.6% ± 4.1%) and 0.01% CHG (76.3% ± 8.6%) causing a decrease in chondrocyte viability compared with controls (97.5% ± 0.6%; *P* < .001 for both). CHG exposure at either concentration had no significant effect on collagen content, while 0.05% CHG exposure led to a significant decrease in mean GAG per wet weight compared with the control group (2.6% ± 1.7% vs 5.2% ± 1.9%; *P* = .029). There was a corresponding weakening of mechanical properties in explants treated with 0.05% CHG compared with controls, with decreases in mean aggregate modulus (177.8 ± 90.1 kPa vs 280.8 ± 19.8 kPa; *P* < .029) and shear modulus (102.6 ± 56.5 kPa vs 167.9 ± 16.2 kPa; *P* < .020).

**Conclusion:**

One-minute exposure to CHG for articular cartilage explants led to dose-dependent decreases in chondrocyte viability, GAG content, and compressive mechanical properties. This raises concern for the risk of mechanical failure of the cartilage tissue after CHG exposure.

**Clinical Relevance:**

Clinicians should be judicious regarding the use of CHG irrigation at these concentrations in the presence of native articular cartilage.

Antiseptic irrigation solutions have gained popularity in recent years for bacterial decontamination in orthopaedic surgeries. These solutions have been used to prevent bacterial growth after joint arthroplasty and within contaminated wounds in the setting of open fractures.^9,20,21^ Although several studies have demonstrated that chlorhexidine gluconate (CHG) solutions can result in catastrophic chondrolysis of articular cartilage,^7,24^ CHG irrigation is still used by some surgeons as a disinfectant during joint surgery in the presence of intact articular cartilage. CHG can come into contact with native articular cartilage if used in such procedures as partial joint replacement, including hip and shoulder hemiarthroplasty, as well as unicompartmental knee arthroplasty and periarticular fracture fixation. A case report and review conducted by Douw et al^7^ examined the effects of CHG irrigation during arthroscopy. In this review, 5 young patients underwent irrigation of the knee with 1% aqueous chlorhexidine during arthroscopy. Two to 3 months after surgery, all these patients had pain, swelling, loss of function, and crepitus, and postoperative radiographs revealed loss of joint space and loose bodies due to extensive chondrolysis.^7^

In contrast, Best et al^3^ showed that a 1-minute exposure to 0.05% CHG did not have adverse effects on nonosteoarthritic cartilage, while decreases in metabolic activity were only observed in osteoarthritic cartilage. Similarly, in a rat patellar model, a 1-minute exposure of 0.05% CHG and jet lavage did not alter cartilage metabolism in vitro, and a 30-minute exposure with or without rinsing produced no impairment of metabolic activity 6 weeks later in vivo.^22^ However, longer exposure times did result in significant decreases in cartilage metabolic activity.^22^ These results suggest that there may be a dose-dependent and exposure time-dependent relationship between CHG exposure and the resultant cellular effects within articular cartilage. Furthermore, CHG solutions have been shown to be an effective disinfectant for accidental contamination of anterior cruciate ligament grafts and osteochondral allografts without affecting their mechanical properties and viability, respectively, suggesting that these grafts can be implanted in the native knee after CHG treatment.^5,10,15,23^

It is currently unknown whether low-dose CHG irrigation solutions used for brief exposures have any detrimental effects on native articular cartilage. Therefore, this study aimed to evaluate the in vitro effects of a 1-minute exposure of CHG solution on native porcine articular cartilage explants. Explants were bathed in various low concentrations (0.05% and 0.01%) of CHG solutions for 1 minute, followed by quantification of chondrocyte viability, biochemical content, and mechanical properties at 7 days after CHG exposure. It was hypothesized that a 1-minute CHG exposure at both concentrations would decrease cell viability, biochemical content, and mechanical properties of articular cartilage explants.

## Methods

### Explant Harvest and CHG Exposure

Disposable biopsy punches, 2.5 mm in diameter, were used to extract osteochondral specimens. Full-thickness articular cartilage punches were harvested from the femoral condyles of 2 juvenile porcine stifle joints <24 hours after slaughter using an aseptic technique (Sierra for Medical Science). To ensure precision, a scalpel was used to cut at the tidemark, approximately 2 mm in thickness, leaving the articular surface undisturbed and unaltered by the biopsy procedure. Any subchondral bone was removed and excluded from the explant culture and any subsequent analyses. The porcine stifle and articular cartilage are widely used for cartilage repair models because of their similarity in mechanical loading profiles and biochemical properties to the human knee and articular cartilage.^17,19^ All cadaveric specimens were grossly normal without any abnormalities of the articular cartilage. Explants were maintained in a chondrogenic medium (Dulbecco’s modified Eagle medium with high-glucose/GlutaMAX containing 1% penicillin/streptomycin/fungizone (PSF), 1% [vol/vol] Insulin-Transferrin-Selenium-Plus premix 1% [vol/vol] nonessential amino acids, 100 nM dexamethasone, 40 µg/mL l-proline, 50 µg/mL ascorbate-2-phosphate, and 100 µg/mL sodium pyruvate; all from Sigma) until CHG exposure. Dexamethasone was added to the chondrocyte cell culture to preserve cell phenotype and enhance functional properties.^12^ On the same day as the harvest, articular cartilage explants (6 per group) were bathed in a CHG solution (0.05% CHG in sterile water; Irrisept; Irrimax Corporation) at varying concentrations (0% control, 0.01% CHG, and 0.05% CHG) for 1 minute. This was followed by 2 rounds of thorough phosphate-buffered saline wash to prevent CHG from interacting with explant medium additives or ingredients. This method and exposure time are analogous to the surgical technique that is recommended by the manufacturer of the irrigation solution for use in surgery.^14^ Explants were then maintained in chondrogenic media at 37°C and 10% CO_2_ for 7 days until testing. Furthermore, different explants were used for various biochemical tests, live/dead assay, histological assessment, and indentation testing. With the exception of the live/dead assay, all specimens were promptly frozen after the culture period and thawed immediately before testing. This protocol was followed to ensure uniform storage conditions and timing for all samples.

### Viability Assessment

Seven days after CHG treatment, explants were incubated in a mixture of 80 µL of chondrogenic medium and 80 µL of LIVE/DEAD reagent (calcein acetoxymethyl, ethidium homodimer-1; Thermo Fisher) for 30 minutes. The explant surfaces were then viewed under fluorescence microscopy using Texas red and green fluorescent protein filters at ×20 magnification. Images were analyzed with the ImageJ software (National Institutes of Health), where 3 regions of interest measuring 150 mm × 150 mm were randomly taken from nonoverlapping areas. Live and dead cells were counted in ImageJ to calculate the viability. An average was taken from 3 regions of interest to obtain 1 measurement of viability per explant.^4,18^

### Histological Evaluation

Explant samples were fixed in 10% neutral buffered formalin, embedded in paraffin, and cut into 4 mm–thick sections. These sections were then stained using hematoxylin and eosin to study cellular morphology, picrosirius red to examine total collagen distribution, and safranin O to analyze the distribution of glycosaminoglycans (GAGs).

### Quantitative Biochemistry

Explants were dabbed dry and immediately weighed to obtain their wet weights (WWs). Then, they were lyophilized and weighed again to obtain their dry weights (DWs). The water content of each explant was calculated using the weight measurements before and after drying. The lyophilized explants were then digested in 125 mg/mL papain solution at 60°C for 18 hours. The content of sulfated GAGs was determined using the Blyscan dimethyl methylene blue assay kit (Biocolor Ltd), and collagen content was quantified using a modified colorimetric chloramine-T hydroxyproline assay with a Sircol collagen assay standard (Biocolor Ltd). DNA content was measured using the PicoGreen cell proliferation assay (Quant-iT PicoGreen dsDNA assay kit; Thermo Fisher). The collagen and GAG content were normalized to WW and DW.^4,18^

### Creep Indentation Testing

A creep indentation apparatus machine was used to assess the viscoelastic compressive properties of explants that had been frozen for 2 weeks in protease inhibitor.^1^ ImageJ was used to measure the thickness of the specimens by graphically scaling the thickness of the sample to a ruler underneath the sample. A 1.0 mm–diameter, flat-ended, porous indenter tip was applied to the samples with loads of 5.05, 7.5, and 12.5 g. The indentation was performed on unconfined cartilage, and samples were allowed to creep until reaching equilibrium, resulting in a strain of approximately 10%, as previously described.^11^ The aggregate modulus and shear modulus were calculated from the resulting experimental data using a standard linear solid model, in conjunction with the measured Poisson ratio, as previously described.^2,16^

### Statistical Analysis

Statistical analysis was carried out using Prism 9 (GraphPad Software). Based on a previous study,^6^ the minimum sample size was determined to be 6 per group, with viability as the primary outcome, an alpha level set at .05, and a minimum power of 80%. A 1-way analysis of variance with Tukey post hoc tests was applied to identify any differences in the effects of CHG dose for all quantitative data. The data are presented as mean ± SD in all bar graphs.

## Results

### Chondrocyte Viability

**Figure 1.**
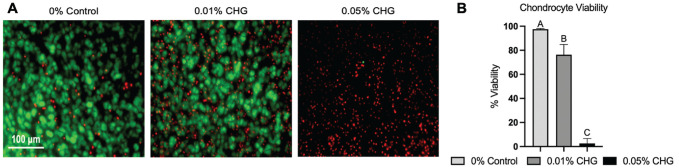
Chondrocyte viability after chlorhexidine gluconate (CHG) exposure. (A) Articular surfaces imaged with live/dead assay (×20) show live (green) and dead (red) chondrocytes. (B) CHG exposure resulted in a decrease in chondrocyte viability. Statistical significance (*P* < .05) among groups is indicated by groups marked with different letters.

Seven days after CHG exposure, a decrease in mean chondrocyte viability in the CHG groups was observed (Figure 1). Representative images of the live/dead assay show an abundance of red (dead) cells and few green (live) cells in the 0.05% CHG–treated explants compared with other groups, and the 0.01% CHG–treated explant had a mix of both live and dead cells. There was a dose-dependent decrease in chondrocyte viability, with 0.05% CHG–treated specimens have the lowest viability (2.6% ± 4.1%, *P* < .001, vs 0.01% CHG and control), followed by 0.01% CHG (76.3% ± 8.6%, *P* < .001, compared with control). Control specimens demonstrated high chondrocyte viability (97.5% ± 0.6%).

### Histology

**Figure 2.**
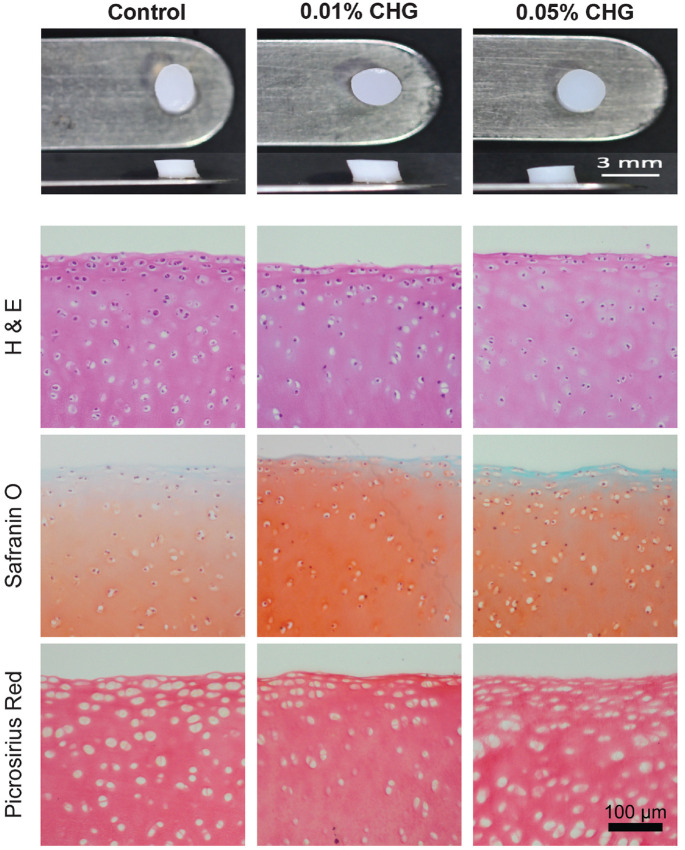
Gross and microscopic histology of explants after exposure to chlorhexidine gluconate (CHG) solution: hematoxylin and eosin (H&E) for cellular morphology, picrosirius red to examine total collagen distribution, and safranin O to analyze the distribution of glycosaminoglycans.

Evaluation of cell morphology showed lower cellularity in the 0.05% CHG–treated group compared with the 0.01% CHG–treated and control groups (Figure 2). GAG and collagen distribution did not appear to be affected by CHG exposure (Figure 2).

### Quantitative Biochemistry

**Figure 3.**
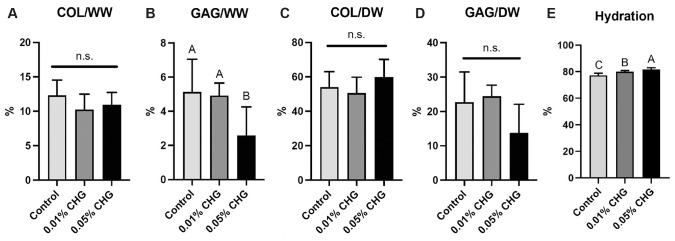
Chlorhexidine gluconate (CHG) did not affect collagen (COL) per wet weight (WW), while 0.05% CHG exposure led to a decrease in glycosaminoglycan (GAG) per WW compared with other groups. Moreover, CHG did not affect collagen per dry weight (DW) or GAG per DW. Statistical significance (*P* < .05) among groups is indicated by groups marked with different letters. n.s., nonsignificant.

The mean collagen content per WW or DW did not differ significantly among groups (Figure 3). However, exposure to 0.05% CHG led to a decrease in mean GAG per WW compared with the negative control (2.6% ± 1.7% vs 5.2% ± 1.9%; *P* = .029) and 0.01% CHG (5.0% ± 0.7%; *P* = .046) (Figure 3B). There were no significant differences in GAG content between the control and 0.01% CHG–treated groups.

### Compressive Mechanical Properties

**Figure 4.**
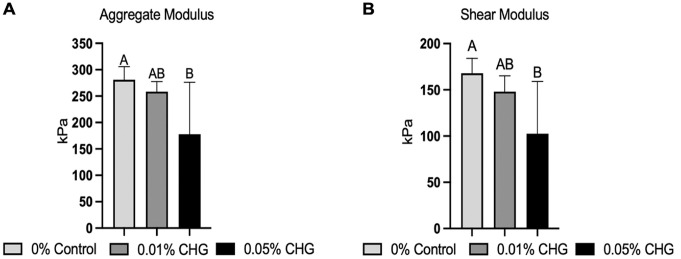
Compressive mechanical properties of articular cartilage explants. Chlorhexidine gluconate (CHG)–exposed explants exhibited a dose-dependent decrease in (A) aggregate modulus and (B) shear modulus. Statistical significance (*P* < .05) among groups is indicated by groups marked with different letters.

A decrease in mean aggregate modulus was seen with CHG exposure. The 0.05% CHG–treated specimens (177.8 ± 90.1 kPa) had a significantly lower mean aggregate modulus compared with the control group (280.8 ± 19.8 kPa) (*P* < .029) (Figure 4). The mean aggregate modulus of the 0.01% CHG group (258.6 ± 19.91 kPa) was not significantly different compared with those groups. CHG exposure also led to a decrease in shear modulus. The 0.05% CHG–treated specimens (102.6 ± 56.5 kPa) had a lower mean shear modulus than the control group (167.9 ± 16.2 kPa) (*P* < .020) (Figure 4). The mean shear modulus of the 0.01% CHG group (148 ± 14.4 kPa) was not significantly different compared with either group.

## Discussion

In this in vitro study, a brief 1-minute exposure of CHG irrigation solution to native articular cartilage explants resulted in dose-dependent chondrotoxicity and changes in extracellular matrix (ECM) composition and mechanics at 7 days. At a dose of 0.05% CHG, which is used clinically in irrigation solutions for bacterial decontamination,^14^ substantial chondrocyte death, GAG loss, and weakening of compressive mechanical properties of the articular cartilage tissue were observed. These findings raise concern for use of CHG irrigation in the presence of native articular cartilage.

The findings of this study suggest that the chondrocyte death results in changes to the ECM composition via a decrease in GAG production during the first 7 days after CHG exposure. The corresponding decrease in GAG content is consistent with the observed decline in the compressive mechanical properties of the explants. A decrease in mean aggregate modulus and shear modulus was observed after CHG exposure, indicating a weakening of the cartilage’s compressive mechanical properties. This weakening would then predispose the articular cartilage to mechanical failure and delamination when subjected to compressive forces, which is consistent with the chondrolysis that has been observed clinically.^7,24^ Although CHG exposure did not have a significant effect on collagen content in the short term, the long-term effects of CHG on collagen turnover are still unknown. Collagen turnover is rather slow in healthy articular cartilage, but if there is substantial chondrocyte death, new collagen would not be synthesized, and the existing collagen may be degraded by an increase in matrix metalloproteinases and shift of cartilage homeostasis to a catabolic state. These changes may eventually result in collagen depletion in the ECM over time, leading to further weakening of the biomechanical properties of the cartilage tissue.^8,13^

To date, the effects of low-dose CHG irrigation on native articular cartilage have not been well studied. Some groups have reported that 0.05% CHG irrigation does not negatively affect articular cartilage when a brief exposure time is used.^3,22^ As in this study, these groups performed a 1-minute exposure of 0.05% CHG to human and rat articular cartilage in vitro. However, in these studies, endpoints were collected immediately after CHG exposure, and only metabolic activity was measured, without attention to cell viability and mechanics.^3,22^ In human articular cartilage, Best et al^3^ still found a 14% to 43% reduction in metabolic activity after a 1-minute exposure to 0.05% CHG. In the present study, the explants were incubated in chondrogenic medium for 7 days after CHG exposure to allow for any potential cellular and biochemical changes to develop. When compared with controls, detrimental effects to chondrocyte viability, GAG content, and compressive mechanics were observed. This is consistent with a study by Campbell et al,^5^ who showed significant cell death in human osteochondral plugs within 1 to 2 days after CHG pulse lavage for concentrations >0.002%. Together, the data support that chondrotoxicity and mechanical weakening can occur even with a 1-minute exposure to low-dose CHG solution, raising concern for its use in the setting of native articular cartilage.

This study has several limitations. First, our creep indentation analysis had a tip-to-sample diameter ratio of 2.5 rather than ≥3, which would be ideal for indentation theory. Second, the in vitro conditions may not accurately reflect in vivo conditions with regard to dosage, exposure time, and clearance from the joint. Blood and synovial fluid may dilute the CHG during surgery, decreasing the risk of chondrotoxicity. Moreover, this study employed juvenile porcine cartilage tissue rather than mature cartilage tissue, which exhibits fewer chondrocytes and a higher proportion of ECM components. However, we expect the same results to apply in mature cartilage tissues. This anticipation arises from the understanding that the consequences of CHG-induced cell death, as well as its disturbance of the ECM by reducing GAG content, are likely to undermine the structural integrity of cartilage. These effects are anticipated to be consistent across different cartilage tissues. However, additional studies should be done to verify this. Third, the live/dead assay in this study only examined cellularity and cell death in the tissue’s outermost layer (superficial tangential zone) up to a depth of approximately 0.2 mm. We encountered challenges when trying to investigate the deeper tissue layers, as the live/dead dye did not penetrate effectively. Because sensitivity to CHG may vary among the different depths from diffusion, we do not know if cell death is consistent throughout the entire thickness of the tissue. Fourth, the removal of the subchondral bone from the native explants created a nonphysiologic interface and increased surface area exposure, which may have increased the penetration of CHG compared with a surface lavage. Furthermore, the location of explants was not considered in the various tests that were performed. Each analysis used a mixture of samples from around the condyle.

Finally, this study examined the viability, biochemical, and mechanical properties 7 days after exposure in an attempt to allow chondrocyte viability to reach a steady state, which may be a limited recovery time for chondrocytes and does not capture the time-dependent effects of CHG on chondrocyte viability thoroughly. It may be possible that later time points would show recovery of GAGs and compressive mechanical properties. Furthermore, our experiment lacked metabolic studies; hence, it is not currently clear how CHG can affect matrix synthesis and metabolic processes of the tissue. Despite these limitations, this study provides insight into the effects of a 1-minute CHG exposure on native cartilage tissues and raises concern for the use of CHG irrigation solution in the setting of native articular cartilage.

## Conclusion

One-minute CHG exposure to articular cartilage explants led to dose-dependent decreases in chondrocyte viability, GAG content, and compressive mechanical properties at 7 days. These detrimental effects were observed for both 0.05% and 0.01% CHG concentrations. The findings of this study suggest increased catabolism after cell death, raising concern for the risk of mechanical failure of the cartilage tissue. Clinicians should be judicious regarding the use of CHG irrigation at these concentrations in the presence of native articular cartilage.
